# Diffuse maculopapular rash: A family cluster during the last Chikungunya virus epidemic in Italy

**DOI:** 10.1002/ccr3.1831

**Published:** 2018-10-22

**Authors:** Silvia Spoto, Elisabetta Riva, Marta Fogolari, Eleonora Cella, Sebastiano Costantino, Silvia Angeletti, Massimo Ciccozzi

**Affiliations:** ^1^ Internal Medicine Department University Campus Bio‐Medico of Rome Rome Italy; ^2^ Unit of Virology University Campus Bio‐Medico of Rome Rome Italy; ^3^ Unit of Clinical Laboratory Science University Campus Bio‐Medico of Rome Rome Italy; ^4^ Unit of Medical Statistics and Molecular Epidemiology University Campus Bio‐Medico of Rome Rome Italy

**Keywords:** CHIKV infection, infection control, maculopapular rash, outbreak, preventive measures

## Abstract

A family cluster of father, mother, and daughter with Chikungunya virus (CHIKV) infection was diagnosed during last epidemic in Italy. In temperate area, during the summer season, clinicians should consider CHIKV infection in the differential diagnosis of patients with fever, maculopapular rash, polyarthralgia, and conjunctival erythema.

## 1 INTRODUCTION

In Kimakonde language, the term Chikungunya means “to become contorted” in reference to the stooped posture assumed by the patient consequently to the joint pain.^1^

Chikungunya is a mosquito‐borne viral disease caused by a RNA virus belonging to the alphavirus genus of the family Togaviridae described in over 60 countries in Asia, Africa, Europe, and the Americas. In the summer 2007, the first autochthonous epidemic outbreak of CHIKV in Europe, in the Region of Emilia‐Romagna of the northeast Italy, was described.^2^

The virus is transmitted by the bites of infected female of the species *Aedes albopictus* in Asia, America, and Europe and *Aedes aegypti* in the tropical and subtropical regions of the world. After the bite of an infected mosquito, the incubation period ranges between 4 and 8 days but can vary from 2 to 12 days (https://www.who.int/mediacentre/factsheets/fs327/en/). During this phase, the chances of transmission from the human to vector are very high.^3^

The illness, asymptomatic in 3%‐28% of cases, can present with an acute auto‐limiting viraemic phase and in some cases with a chronic postinfectious inflammatory phase.^4^

The acute infection is characterized by the sudden onset of the following symptoms: high fever (>39°C; almost 80%); bilateral symmetrical polyarthralgia, involving peripheral and larger joints (87%‐98%) especially at hands (50%‐76%), wrists (29%‐81%) and ankles (41%‐68%); myalgia without myositis (46%‐59%); ligament pain, tenosynovitis; macular or maculopapular pruriginous rash involving mainly the limbs, trunk, and face (20%‐80%); nausea, vomiting, and abdominal pain (15%‐47%).

Atypical symptoms including conjunctivitis, neuroretinitis, iridocyclitis, myocarditis, pericarditis, pneumonia, dry cough, lymphadenopathy, nephritis, hepatitis, digestive alteration, and pancreatitis have been described.^5^

Mortality rates are very low, and it has been reported especially among elderly and infants due to severe forms including heart failure, acute myocardial infarction, toxic hepatitis, encephalitis, bullous dermatosis, pneumonia, renal failure, and sepsis with multiple organ failure syndrome.^6^

Abnormal laboratory findings such as anemia, lymphopenia, moderate thrombocytopenia and leucopenia, elevated liver enzymes, creatinine, creatinine kinase, and hypocalcemia have also been reported. During the chronic phase, high levels of C‐reactive protein and persistent IgM and IgG anti‐chikungunya antibodies are observed.^7^

After infection, the innate immune system controls CHIKV replication that is cleared within 4‐7 days. On the contrary, the adaptative immune response (CHIK‐specific B cell and T‐cell activation) requires about a week to develop.^8^

The chronic postinfectious inflammatory phase is characterized by arthralgia mostly monoarticular, oligoarticular, rarely polyarticular lasting for weeks, months, or years; persisting myalgia at the arms, ligament or tendon inflammation (Achilles tendonitis, plantar fasciitis), fibromyalgia and rarely by rash, alopecia, pruritus, bilateral Raynaud phenomenon, digestive and cerebral disorders, dysesthesia, and paraesthesia.

During the chronic phase in more than 5% of cases, the modified version of the American College of Rheumatology (ACR) criteria for rheumatoid arthritis (RA) are satisfied, even 27.5 months after infection. For this reason, the differential diagnosis with RA, spondyloarthropathy, undifferentiated or psoriatic arthritis, or reactivation of previous osteoarthritis is needed.^9^ In endemic zone, the differential diagnosis of dengue infection (hemorrhagic fever, retro‐orbital headache, arthralgia, myalgia, nausea, and vomiting) is necessary. Support therapy is efficacy in the most of the cases.

## 2 FAMILY CLUSTER

**Figure 1:**
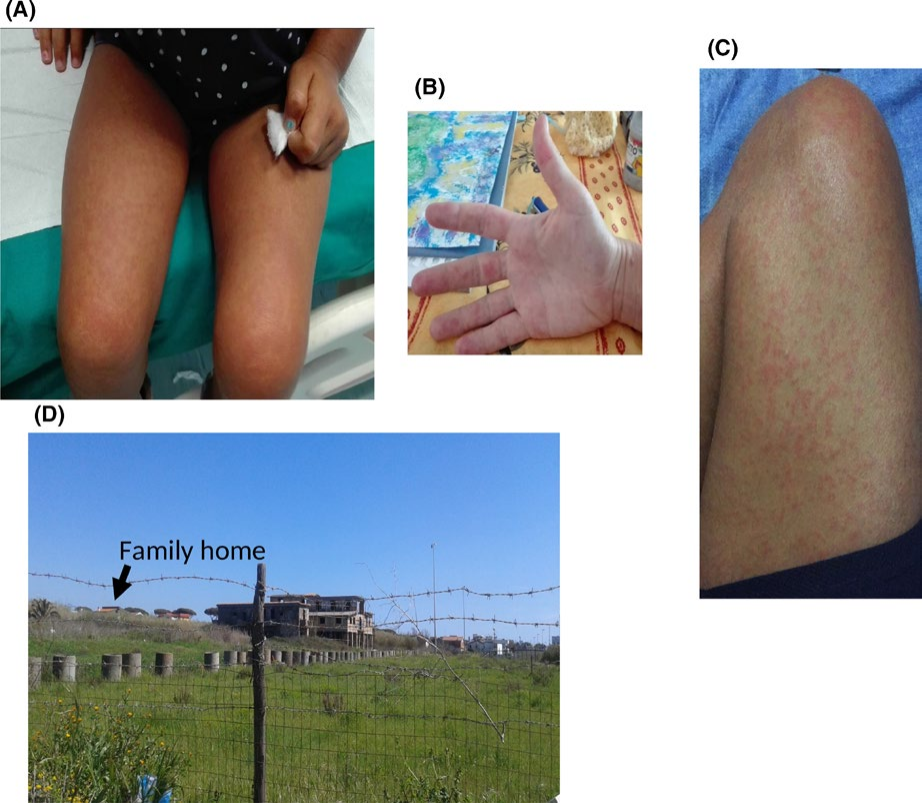
Daughter’s cutaneous maculopapular rash at inferior limbs (A), Mother’s cutaneous maculopapular rash at superior limbs (B) and father’s cutaneous maculopapular rash at inferior limbs (C). Abandoned building surrounded by containers full of stagnant water extending for about 2 km including the home area distant about 200 m indicated by the arrow (D)

A family composed of father of 34 years old, mother of 34 years old, and daughter of 3 years old was in good health status until August 2017, during their holydays in Anzio, a sea location near Rome, Italy, when suddenly get sick. First, the daughter presented fever (40°C) for 3 days and appearance of cutaneous maculopapular rash at trunk, inferior and superior limbs and conjunctival erythema after fever defervescence, lasting 24 hours (Figure 1A). After 1 week, first the mother and 2 days later the father, developed high fever (>40.5°C) for 3 days, the same maculopapular rash with eruption at the hands (Figure 1B,C), itching conjunctival erythema, polyarthralgia involving peripheral joints of hands, feet, wrists, ankles and cervical region with functional disability, asthenia, abdominal discomfort and respiratory fatigue, following fever defervescence. Spontaneous regression of fever and rash was observed after 3 and 5 days from their appearance, respectively.

Fifteen days from symptoms onset, for the persistence of the conjunctival erythema in the daughter and of the polyarthralgia in the parents, a physician consultation was required.

Cardio‐pulmonary, abdominal, lymphonodal, and neurological examinations were unremarkable. No signs of inflammation were evident at joints. The laboratory data including blood count cells and C‐reactive protein (CRP) were in normal range (Table 1). The contemporary description of cases of CHIKV infection registered in the same area made suppose the presence of this infection. The diagnosis was confirmed by the presence of IgM and IgG anti‐chikungunya antibodies by Enzy‐well Chikungunya (IgG and IgM kit) qualitative ELISA test (Diesse, Siena, Italy), 15 days after the potential contagion. IgG antibodies resulted positive in all three patients, whereas IgM tested positive only in the father, the most recently infected (Table 1). The virus was not isolated from the blood in anyone of them, because it can be found only during the first few days of infection. Twenty days from symptoms onset, conjunctival erythema disappeared, while polyarthralgia of the peripheral joints with mild functional disability persisted in the parents for more than 2 months showing a very slow improvement despite paracetamol assumption.

**Table 1:** Laboratory data of the family cluster

Variable	Reference range	Daughter	Mother	Father
Hematocrit (%)	(women) 36‐46; (man) 36‐46; (child) 28‐42	37.8	36.8	42.3
Hemoglobin (g/dL)	g/dL (women) 12‐16; (man) 13.5‐17.5; (child) 10.5‐15.5	12.4	11.9	14.2
White blood cell (WBC) number *10^3^/mm^3^	µL 4.00‐10.00 (women‐man); (child) 5.50‐15.00	10.09	8.15	6.84
Neutrophils (%)	(women‐man) 40‐80 (child) 30‐55	29.5	59	59
Lymphocytes (%)	(women‐man) 20‐40 (child) 40‐57	57.5	31	28
Platelet count (per mm^3^) *10^3^	150.00‐400.00	305	354	297
Erytrocyte number *10^6^/mm^3^	(women‐man) 4.30‐5.50; (child) 3.60‐5.00	4.70	4.32	5.04
C‐reactive protein (PCR) (mg/L)	<3	<3	<3	<3
Ferritin (ng/mL)	(women‐man) 8‐252; (child) 5‐200	35	17	218
Iron (µg/dL)	(women‐man) 50‐170; (child) 53‐119	109	77	100
Aspartate aminotransferase (AST) (U/L)	0‐37	24	6	19
Alanine aminotransferase (ALT) (U/L)	0‐78	16	15	42
Alkaline phospahatase (ALP) (U/L)	(women‐man) 56‐155; (child) 110‐550	167	70	60
Lactate dehydrogenase (LDH) U/L	(women‐man) 0‐248; (child) 150‐500	261	179	203
Anti‐CHKV IgG	Negative	Positive	Positive	Positive
Anti‐CHKV IgM	Negative	Negative	Negative	Positive
Anti nuclear antibodies	Absence	Absence	Absence	Absence
Anti ds‐DNA antibodies	Absence	Absence	Absence	Absence
Extractable nuclear antigen (ENA) antibodies	Absence	Absence	Absence	Absence
Reumathoid factor (RF) (UI/L)	<15	<10	<10	<10

## 3 DISCUSSION

The family cluster with CHIKV infection described in this report was reported during the last outbreak in Anzio, Italy.^1^ The family lived near an abandoned building surrounded by containers full of stagnant water extending for about 2 km including the home area distant about 200 m (Figure 1D). *Aedes albopictus*, highly resistant and easily adapting to climate variations, lays eggs in any water container in different rural or urban environments.^10^ Parents confirmed to not have used insect repellent and to have been resident in the area of Anzio, where last CHIKV outbreak was described, before disease appearance. It is conceivable that this family outbreak was promoted by the absence of preventive measure adoption, as disinfestation toward the endemic vector *A. albopictus* and protection from insect bite.

Preventive measures for vector control using pyrethrins for 3 days, formulations of insect growth regulators, house‐to‐house interventions to eliminate breeding place, contact tracing, active clinical surveillance of close contacts with isolation of suspected patients, rapid report to local health department to avoid epidemics are recommended.^10^ In temperate area during summer season, clinicians should consider CHIKV infection in the differential diagnosis of patients with fever, maculopapular rash, polyarthralgia and conjunctival erythema. In the family cluster, the clinical presentation required the differential diagnosis with other causes of polyarthralgia following fever and cutaneous maculopapular rash such as RA, spondyloarthropathy, undifferentiated and psoriatic arthritis.

World Health Organization (WHO) outlined that for outbreak prevention, controlling the mosquito vector represents the key factor. For vector control, the elimination of breeding sites and all life stages of the *Aedes* mosquito are strongly recommended. At this aim, environmental disinfestation is suggested.

The outbreak of CHIKV infection reported in the summer 2017 at Anzio (Central Italy),^1^ 10 years after the first during the summer 2007 in Emilia‐Romagna (northeastern Italy),^2^ suggests that countries with a temperate climate, where the virus vector is endemic, represents regions at high risk for CHIKV epidemics. These regions are of public health concern, especially when normal measures to fight vectors, as disinfestation, has not been executed in springer season as in the last Italian case.

## CONFLICT OF INTEREST

None declared.

## AUTHOR CONTRIBUTION

SS, SA and MC: wrote and elaborated the manuscript. ER, MF and EC: performed the laboratory testing for the diagnosis. SS and SC: performed the clinical diagnosis and the patients follow‐up. MC and SA: evaluated the epidemiological data. SS, ER, MF, EC, EC, SA and MC: contributed to data analysis, drafting and revision of the manuscript and agree to be responsible for any aspect of the manuscript.
